# Diffuse Large B‐Cell Lymphoma With Pulmonary and Gastric Involvement Mimicking Nontuberculous Mycobacterial Lung Disease

**DOI:** 10.1002/rcr2.70601

**Published:** 2026-04-30

**Authors:** Kosuke Masuda, Kazuhiro Asada, Yuu Saitoh, Makoto Suzuki, Emi Fukasawa, Yumi Fujita, Kosuke Suzuki, Sho Murai, Daisuke Akahori, Mika Saigusa, Taisuke Akamatsu, Akito Yamamoto, Satoru Morita, Toshihiro Shirai

**Affiliations:** ^1^ Department of Respiratory Medicine Shizuoka General Hospital Shizuoka Japan; ^2^ Department of Hematology Shizuoka General Hospital Shizuoka Japan; ^3^ Department of Diagnostic Pathology Shizuoka General Hospital Shizuoka Japan; ^4^ Department of Thoracic Surgery Shizuoka General Hospital Shizuoka Japan

**Keywords:** diffuse large B‐cell lymphoma, *Mycobacterium intracellulare*, nontuberculous mycobacteria, pulmonary nodules, radiological heterogeneity

## Abstract

An 81‐year‐old man was incidentally found to have multiple bilateral pulmonary nodules on chest computed tomography. Bronchoscopy revealed *Mycobacterium intracellulare* , and partial spontaneous regression of several nodules initially supported a diagnosis of nontuberculous mycobacterial lung disease. However, other nodules showed progressive enlargement during antimicrobial therapy. Surgical lung biopsy revealed diffuse large B‐cell lymphoma (DLBCL), and subsequent evaluation demonstrated gastric involvement. The patient achieved complete remission after systemic chemotherapy. This case highlights the diagnostic challenge of pulmonary lymphoma presenting with heterogeneous radiological behaviour and emphasizes the importance of histological confirmation when the clinical course is atypical.

## Graphical abstract

An 81‐year‐old man presented with multiple bilateral pulmonary nodules and detection of *Mycobacterium intracellulare* , initially suggesting nontuberculous mycobacterial lung disease. However, heterogeneous radiological changes and progressive enlargement of selected nodules led to surgical lung biopsy, which revealed diffuse large B‐cell lymphoma with gastric involvement. This case highlights the importance of histological confirmation when imaging findings are discordant.

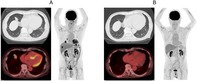

## 1 Introduction

Diffuse large B‐cell lymphoma (DLBCL) involving the lung is uncommon and can mimic various infectious and inflammatory diseases because of its nonspecific radiological features [1, 2]. Nontuberculous mycobacterial lung disease represents a challenging differential diagnosis, since NTM may be isolated from respiratory specimens due to colonization or environmental contamination, and microbiological findings alone do not necessarily indicate active or progressive disease. Therefore, careful correlation of radiological and microbiological findings is essential to avoid diagnostic delay. We report a case of DLBCL with pulmonary and gastric involvement in which concomitant detection of *Mycobacterium intracellulare* and partial radiological regression complicated the initial diagnostic assessment.

## 2 Case Report

**FIGURE 1:**
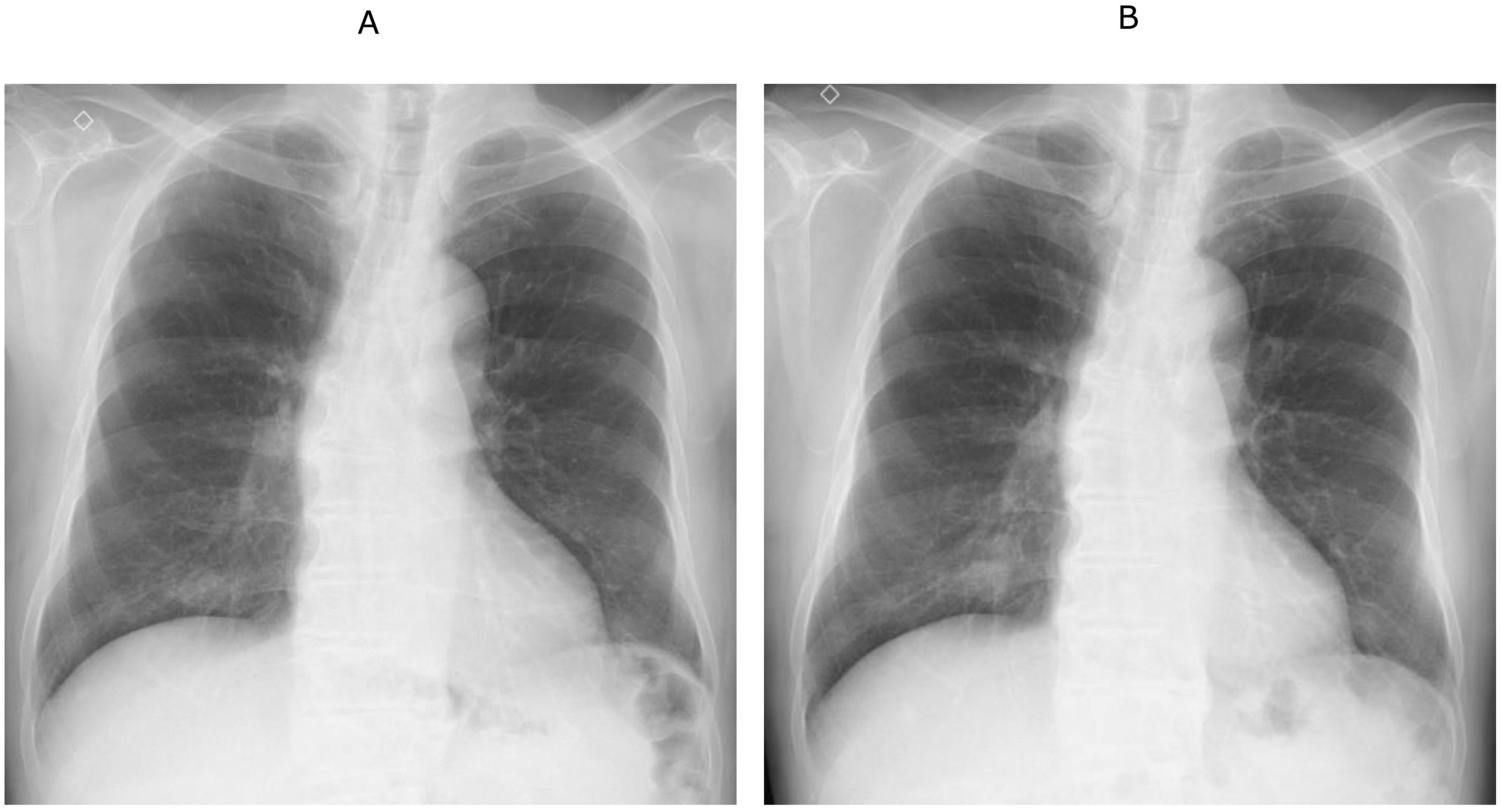
Chest radiographs. (A) Chest radiograph obtained at the initial presentation showing multiple nodular opacities in both lungs, predominantly in the lower lung fields. (B) Follow‐up chest radiograph obtained after the initiation of antimicrobial therapy showing interval changes in the pulmonary nodules, corresponding to the heterogeneous evolution observed on CT.

**FIGURE 2:**
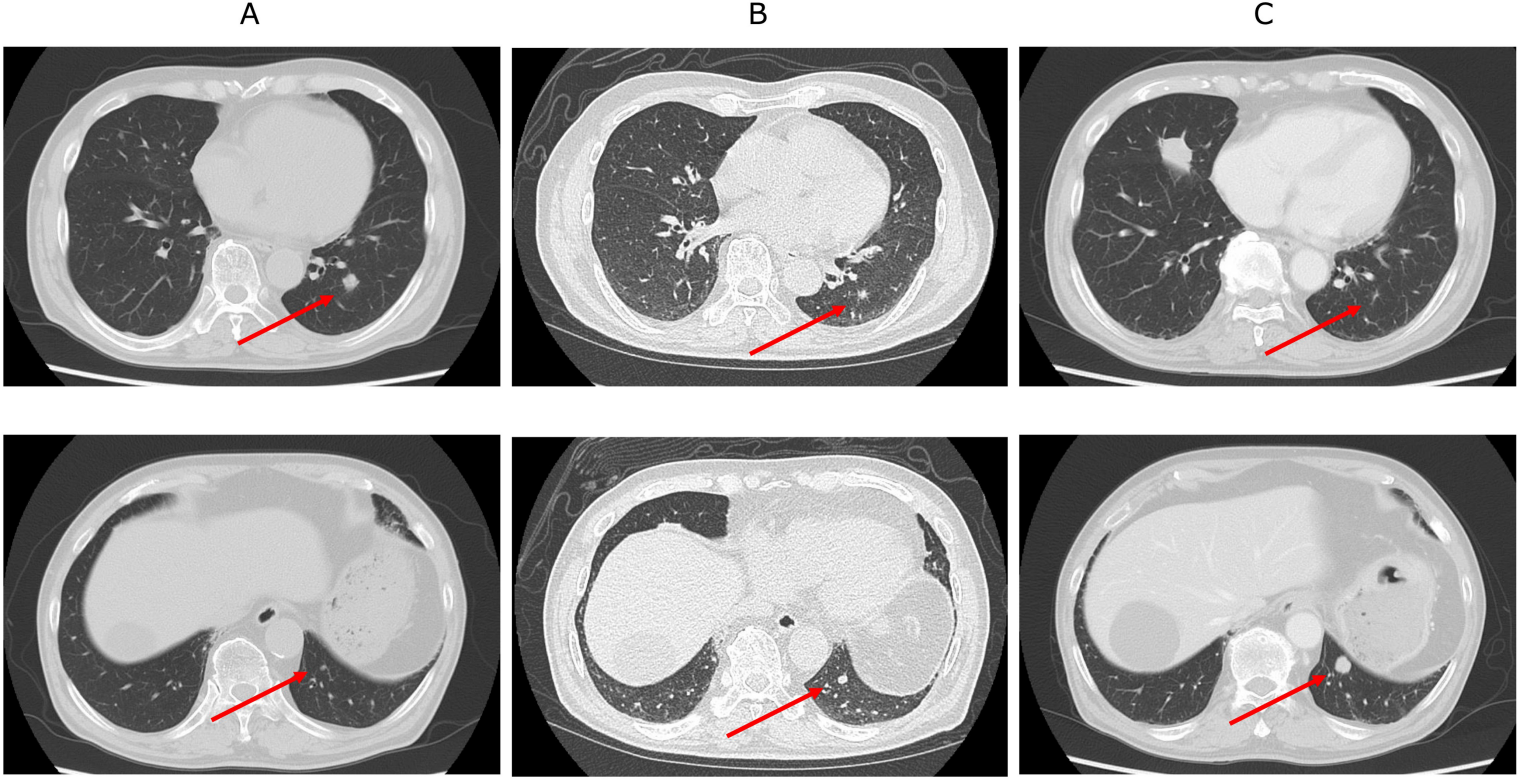
Serial chest computed tomography demonstrating heterogeneous temporal changes of multiple pulmonary nodules. Representative axial images obtained at baseline (A), 2 weeks after the initial CT and before antimicrobial therapy (B), and 8 weeks after the initial CT and after therapy initiation (C). The upper row shows spontaneous regression of one of the multiple pulmonary nodules (arrows), whereas the lower row demonstrates progressive enlargement of another distinct nodule despite antimicrobial therapy; this lesion was subsequently selected for surgical lung biopsy (arrows).

An 81‐year‐old man who had been hospitalized for pulmonary thromboembolism approximately 1 year earlier developed upper gastrointestinal bleeding while receiving anticoagulation therapy, which prompted upper endoscopy. At the time of pulmonary embolism diagnosis, contrast‐enhanced whole‐body computed tomography (CT) revealed no pulmonary nodules or gastric wall abnormalities. Superficial oesophageal cancer was incidentally detected, and staging evaluation was performed. Chest radiography and CT obtained as part of the staging workup demonstrated multiple bilateral pulmonary nodules with relatively smooth margins and no cavitation; the largest nodule measured approximately 30 mm (Figures 1A and 2A).

Five days after the initial CT, the patient was referred to our department for further evaluation.

Laboratory findings at the initial visit revealed a haemoglobin level of 14.3 g/dL, lactate dehydrogenase (LDH) of 181 U/L, and soluble interleukin‐2 receptor (sIL‐2R) of 302.8 U/mL, all within the normal reference ranges.

One week after referral, bronchoscopy with transbronchial lung biopsy (TBLB) demonstrated foamy macrophage accumulation without evidence of malignancy. Polymerase chain reaction analysis of bronchial washing fluid identified *Mycobacterium intracellulare* .

On the CT images acquired simultaneously with positron emission tomography–computed tomography (PET‐CT), performed 2 weeks after the initial CT and before antimicrobial therapy, some pulmonary nodules showed interval regression despite the absence of treatment, whereas others showed no apparent change and persisted, indicating heterogeneous radiological behaviour suggestive of inflammatory lesions (Figure 2B).

**FIGURE 3:**
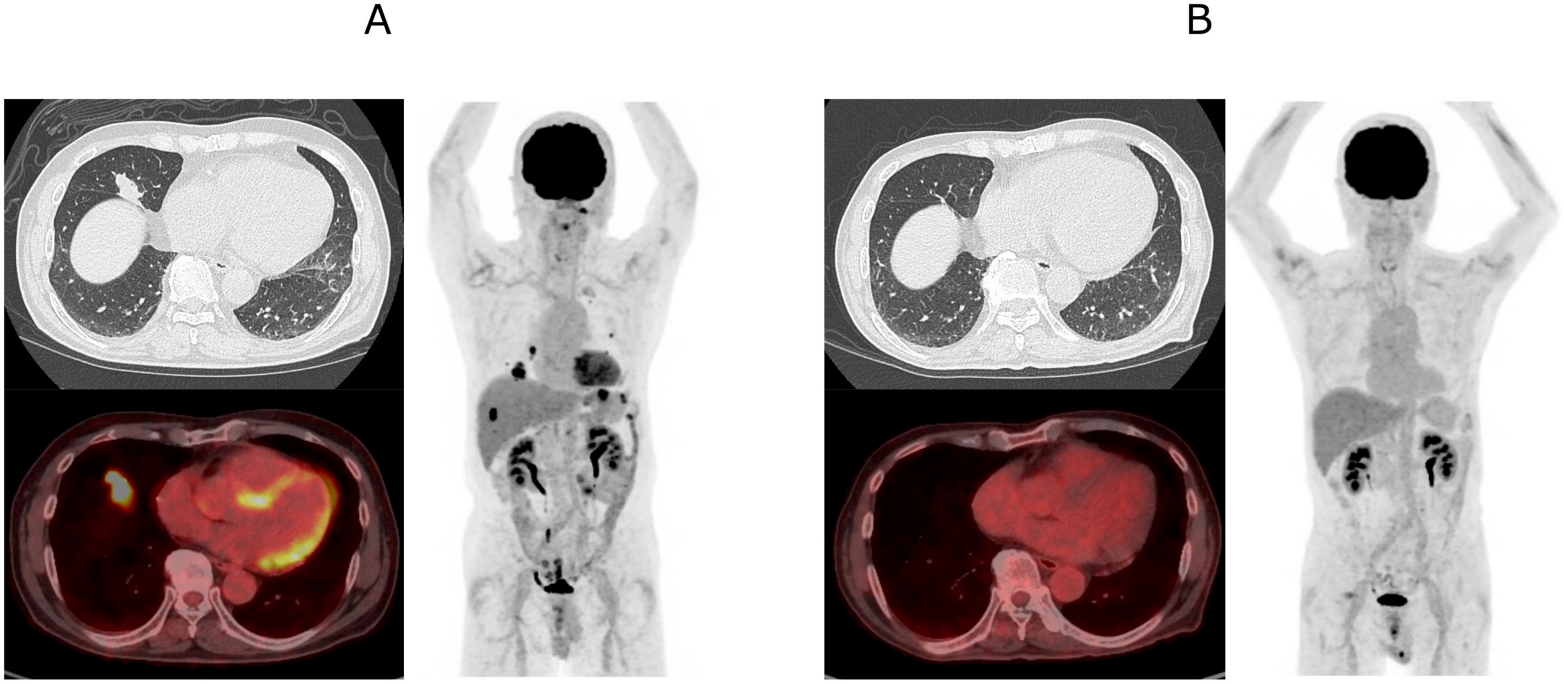
FDG‐PET imaging before and after chemotherapy. (A) Pre‐treatment images showing abnormal FDG uptake in the lung and stomach on fused PET/CT and maximum intensity projection (MIP) images. (B) Post‐treatment images demonstrating complete resolution of abnormal FDG uptake, consistent with a complete metabolic response.

PET‐CT demonstrated fluorodeoxyglucose (FDG) uptake in multiple pulmonary nodules, with a maximum standardized uptake value of 10.55 (Figure 3A).

Based on these findings, the pulmonary nodules and FDG uptake were attributed to *Mycobacterium avium* complex–related inflammatory changes.

Three weeks after bronchoscopy, cultures of bronchial washing fluid confirmed the growth of *Mycobacterium intracellulare* , and the patient was diagnosed with nontuberculous mycobacterial lung disease. Combination antimicrobial therapy with ethambutol and clarithromycin was initiated.

Three weeks after initiation of antimicrobial therapy, follow‐up chest radiography and CT demonstrated further regression or disappearance of several nodules; however, some nodules showed clear and progressive enlargement, indicating that the radiological findings were not fully attributable to nontuberculous mycobacterial infection alone (Figures 1B and 2C). Among these, the nodule located in the left lower lobe showed the most prominent progression and was therefore selected for surgical lung biopsy.

**FIGURE 4:**
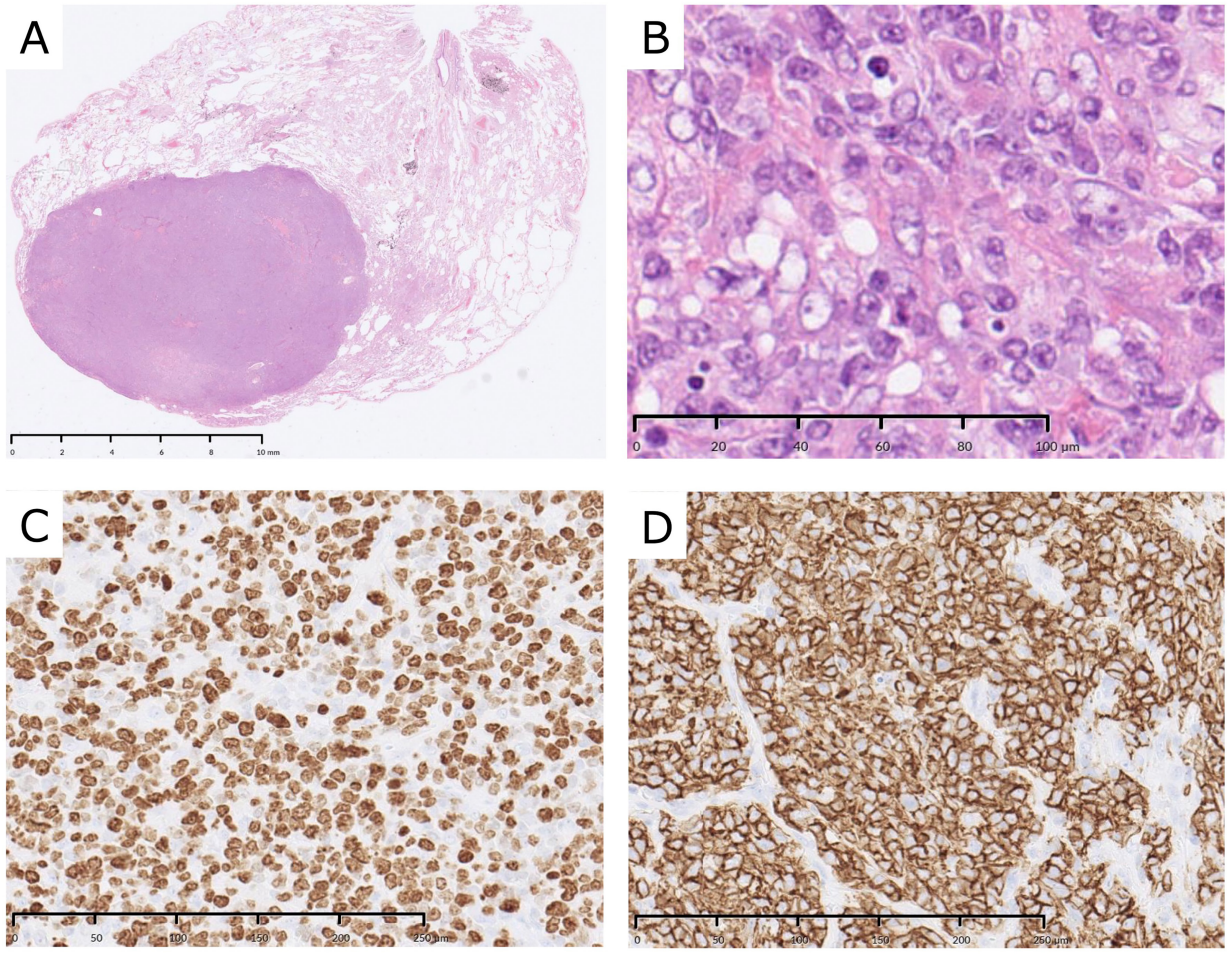
Histopathological findings of the lung biopsy specimen. (A) Low‐power haematoxylin and eosin (H&E) staining showing diffuse infiltration of atypical lymphoid cells. (B) High‐power H&E staining revealing large atypical lymphoid cells with vesicular nuclei and prominent nucleoli. (C) Immunohistochemical staining demonstrating diffuse positivity for CD20. (D) A high Ki‐67 labelling index (approximately 80%), indicating high proliferative activity.

Subsequently, video‐assisted thoracoscopic surgery (VATS) partial resection of the left lower lobe nodule was performed. Histopathological examination revealed diffuse infiltration of large atypical lymphoid cells (Figure 4A,B). Immunohistochemical analysis demonstrated diffuse positivity for CD20 (Figure 4C), positivity for CD10 and BCL6, strong expression of BCL2, and a high Ki‐67 labelling index of approximately 80% (Figure 4D), consistent with DLBCL of germinal centre B‐cell–like phenotype. Epstein–Barr virus in situ hybridization was negative.

Following the diagnosis of DLBCL, we retrospectively reviewed the patient's prior gastrointestinal evaluations. Three upper gastrointestinal endoscopic examinations had been performed between the diagnosis of pulmonary thromboembolism and the patient's presentation to our department, none of which demonstrated findings suggestive of lymphoma. The most recent examination, performed 2 weeks prior to presentation, revealed two ulcer scars in the antrum, and biopsy showed only chronic gastritis.

At the time of the initial CT, no gastric wall abnormalities were observed. PET‐CT performed 2 weeks after the initial CT demonstrated FDG uptake in the gastric wall without corresponding wall thickening. Gastric wall thickening became evident only 8 weeks after the initial CT on follow‐up CT.

Because abnormal FDG uptake in the stomach had also been observed on PET‐CT, upper endoscopy was subsequently performed and revealed a 35‐mm ulcerative lesion. Histological examination of the gastric biopsy specimen confirmed gastric involvement of DLBCL.

The patient was treated with six cycles of polatuzumab‐rituximab‐cyclophosphamide‐doxorubicin‐prednisolone (PR‐CHP). Post‐treatment imaging demonstrated complete resolution of the pulmonary nodules on chest CT and disappearance of abnormal FDG uptake in both the lung and stomach on PET‐CT, indicating a complete response (Figure 3B).

## 3 Discussion

DLBCL rarely presents as multiple pulmonary nodules, and its radiological appearance often overlaps with infectious or inflammatory lung diseases [1, 2, 3].

Pulmonary involvement of lymphoma typically manifests as consolidations, masses, or peribronchovascular infiltrates, whereas presentation as scattered nodules of varying sizes is less common [1, 2]. In addition, cavitation is unusual in pulmonary lymphoma and may help distinguish it from certain infectious aetiologies. In this patient, the absence of cavitation and the discordant temporal changes in nodule size were subtle but important clues suggesting an alternative diagnosis.

In contrast to the typical nodular bronchiectatic pattern of pulmonary NTM disease, characterized by centrilobular nodules, tree‐in‐bud appearance, bronchiectasis, and occasionally cavitary lesions, this case demonstrated multiple bilateral nodules measuring 5–10 mm and a larger 30‐mm nodule without bronchiectasis, cavitation, or tree‐in‐bud findings. These features were atypical for classic NTM lung disease.

In the present case, the detection of *Mycobacterium intracellulare* in bronchial washing fluid, together with partial regression of several nodules observed over a short period without treatment, initially supported a diagnosis of nontuberculous mycobacterial lung disease. However, subsequent follow‐up imaging after the initiation of antimicrobial therapy revealed progressive enlargement of other nodules, representing an atypical and discordant radiological course that ultimately prompted surgical lung biopsy and led to the correct diagnosis.

NTM can be isolated from respiratory specimens, particularly in elderly patients or those with underlying lung disease, and its detection does not necessarily indicate active disease [4]. Current diagnostic criteria for NTM lung disease emphasize the integration of clinical, radiological, and microbiological findings [4]. Although microbiological criteria were fulfilled in this case, the heterogeneous radiological evolution was not entirely consistent with typical NTM lung disease, underscoring the importance of reassessing the diagnosis when the clinical course deviates from expectations.

At initial visit, haemoglobin, LDH, and soluble IL‐2 receptor levels were within normal ranges, providing no laboratory clues suggestive of lymphoma. This absence of typical hematologic abnormalities further complicated early diagnosis.

Another notable feature of this case was the temporal evolution of gastric involvement. Retrospective review confirmed that no gastric wall abnormalities were present on the initial CT, and early endoscopic biopsy demonstrated only chronic gastritis. Although abnormal FDG uptake in the stomach was observed on PET‐CT, it was initially considered nonspecific because upper endoscopy performed approximately 1 month earlier showed no apparent lesions. Gastric wall thickening became evident later in the clinical course. Because lymphoma is a systemic disease that can involve multiple organs, evolving extrapulmonary findings should be carefully re‐evaluated when the overall disease course is discordant with a single diagnosis [5]. Subsequent endoscopic biopsy confirmed gastric involvement of DLBCL, highlighting the systemic nature of the disease.

Pulmonary lymphoma detected during follow‐up for NTM infection has occasionally been reported. However, to our knowledge, cases in which microbiological confirmation of *Mycobacterium intracellulare* contributed to the initial diagnosis and subsequent re‐biopsy revealed DLBCL have not been previously reported.

The relationship between microbiologically confirmed NTM and DLBCL in this case was difficult to determine. Although the diagnostic criteria for NTM lung disease were fulfilled, spontaneous improvement of radiologic findings has been described in untreated NTM lung disease. Spontaneous regression of malignant lymphoma, including DLBCL, has also been reported, albeit rarely [6]. Retrospectively, complete resolution of pulmonary nodules after lymphoma‐directed therapy suggests that DLBCL was the dominant active disease process. However, because antimycobacterial therapy was administered concurrently, coexistence cannot be definitively excluded.

This case highlights the diagnostic challenge posed by pulmonary lymphoma mimicking infectious lung disease, particularly in the presence of microbiological findings suggestive of NTM infection. When pulmonary nodules demonstrate heterogeneous responses to antimicrobial therapy, clinicians should maintain a high index of suspicion for alternative diagnoses, including malignancy, and consider histological confirmation to avoid delays in appropriate treatment.

## Author Contributions

K.M. managed the patient, collected clinical data, and drafted the manuscript. M.S. performed the pathological evaluation. Y.S. managed the lymphoma treatment. S.M. performed the surgical lung biopsy. All authors contributed to data interpretation, critically revised the manuscript, and approved the final version.

## Funding

The authors have nothing to report.

## Consent

The authors declare that written informed consent was obtained for the publication of this manuscript and accompanying images using the consent form provided by the Journal.

## Conflicts of Interest

The authors declare no conflicts of interest.

## Data Availability

Data sharing is not applicable to this article as no datasets were generated or analysed during the current study.
